# Supplementary material for: Multi‐omic rejuvenation of naturally aged tissues by a single cycle of transient reprogramming
Source: Aging Cell. 2022 Mar 2;21(3):e13578. doi: 10.1111/acel.13578 (PMC8920440; doi:10.1111/acel.13578)
Supplement: Supplementary file 1 — Fig S1‐S5 [file ACEL-21-e13578-s009.pdf]

Figure S1

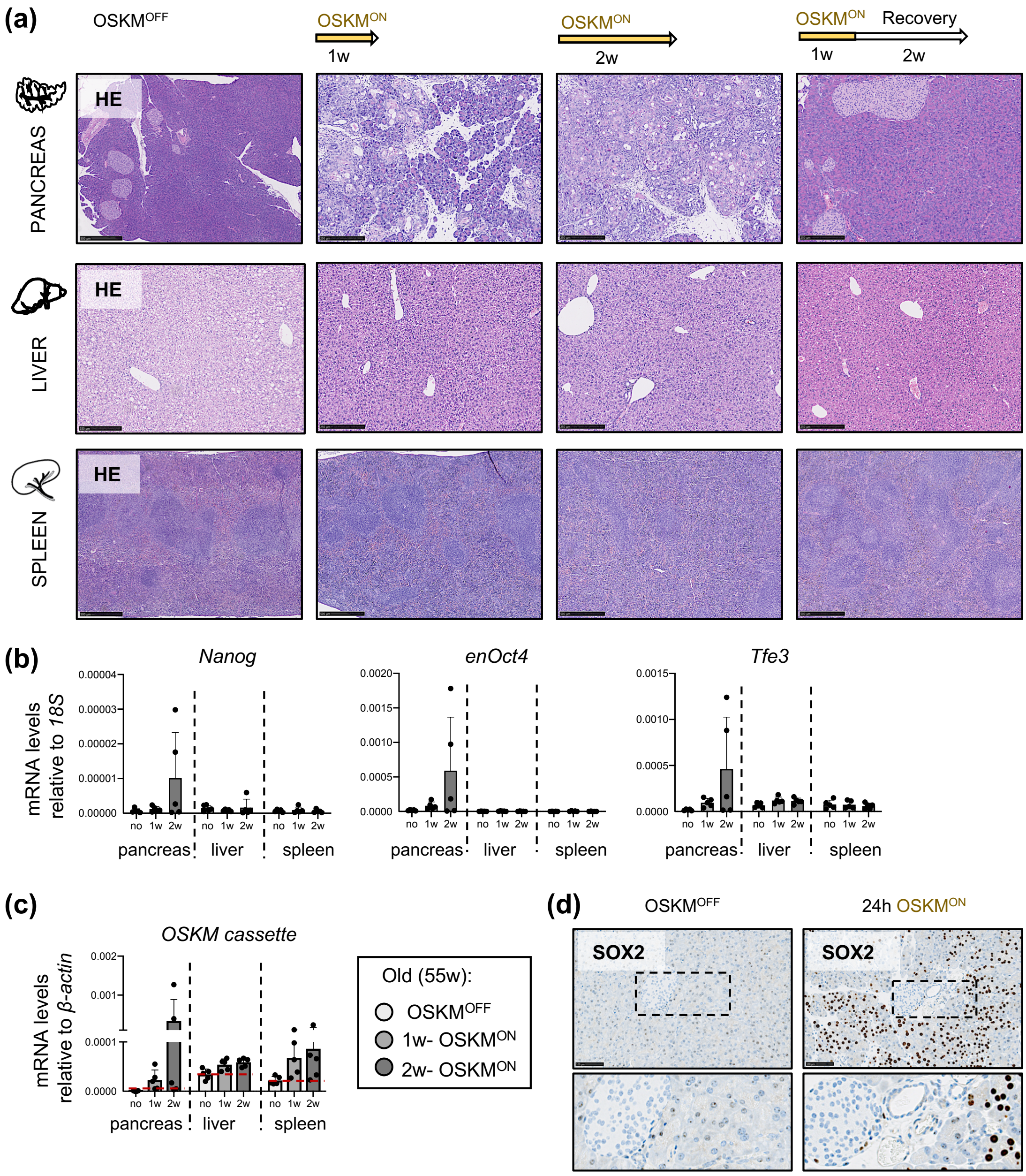

(a)
OSKMOFF
OSKMON
1w
OSKMON
2w
OSKMON
Recovery
1w
2w
PANCREAS
LIVER
SPLEEN
HE
(b)
Nanog
enOct4
Tfe3
mRNA levels relative to 18S
no 1w 2w
pancreas
liver
spleen
(c)
OSKM cassette
mRNA levels relative to β-actin
pancreas
liver
spleen
Old (55w):
OSKMOFF
1w- OSKMON
2w- OSKMON
(d)
OSKMOFF
24h OSKMON
SOX2

Figure S1

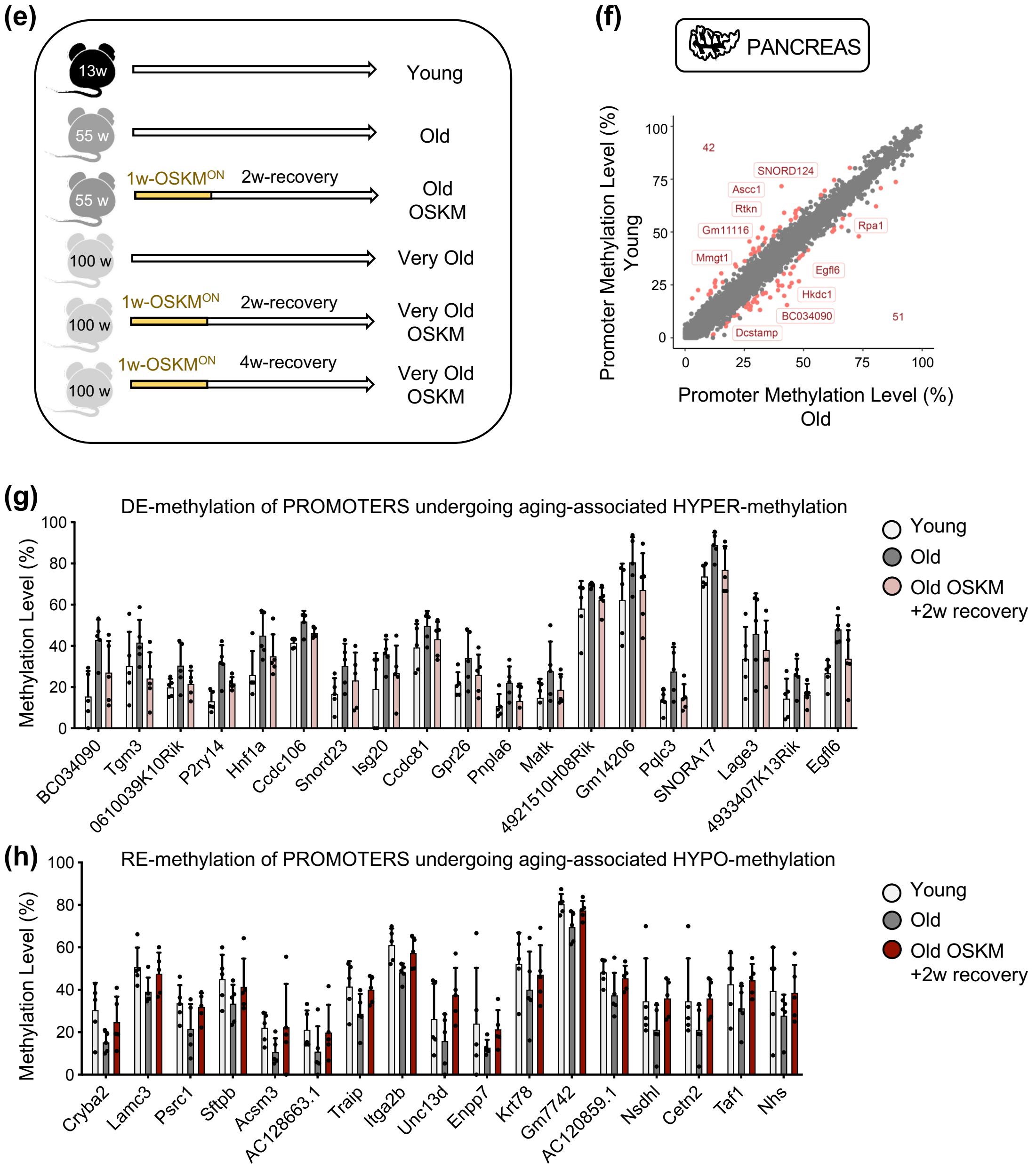
(e)
13w
Young
55 w
Old
1w-OSKM^ON
2w-recovery
Old OSKM
100 w
Very Old
Very Old OSKM
4w-recovery
(f)
PANCREAS
Promoter Methylation Level (%) Young
Promoter Methylation Level (%) Old
42
51
SNORD124
Ascc1
Rtkn
Gm11116
Mmgt1
Rpa1
Egfl6
Hkdc1
BC034090
Dcstamp
(g)
DE-methylation of PROMOTERS undergoing aging-associated HYPER-methylation
Methylation Level (%)
Young
Old
Old OSKM +2w recovery
BC034090
Tgm3
0610039K10Rik
P2ry14
Hnf1a
Ccdc106
Snord23
Isg20
Ccdc81
Gpr26
Pnpla6
Matk
4921510H08Rik
Gm14206
Pqlc3
SNORA17
Lage3
4933407K13Rik
Egfl6
(h)
RE-methylation of PROMOTERS undergoing aging-associated HYPO-methylation
Methylation Level (%)
Young
Old
Old OSKM +2w recovery
Cryba2
Lamc3
Psrc1
Sftpb
Acsm3
AC128663.1
Traip
Itga2b
Unc13d
Enpp7
Krt78
Gm7742
AC120859.1
Nsdhl
Cetn2
Taf1
Nhs

Figure S1

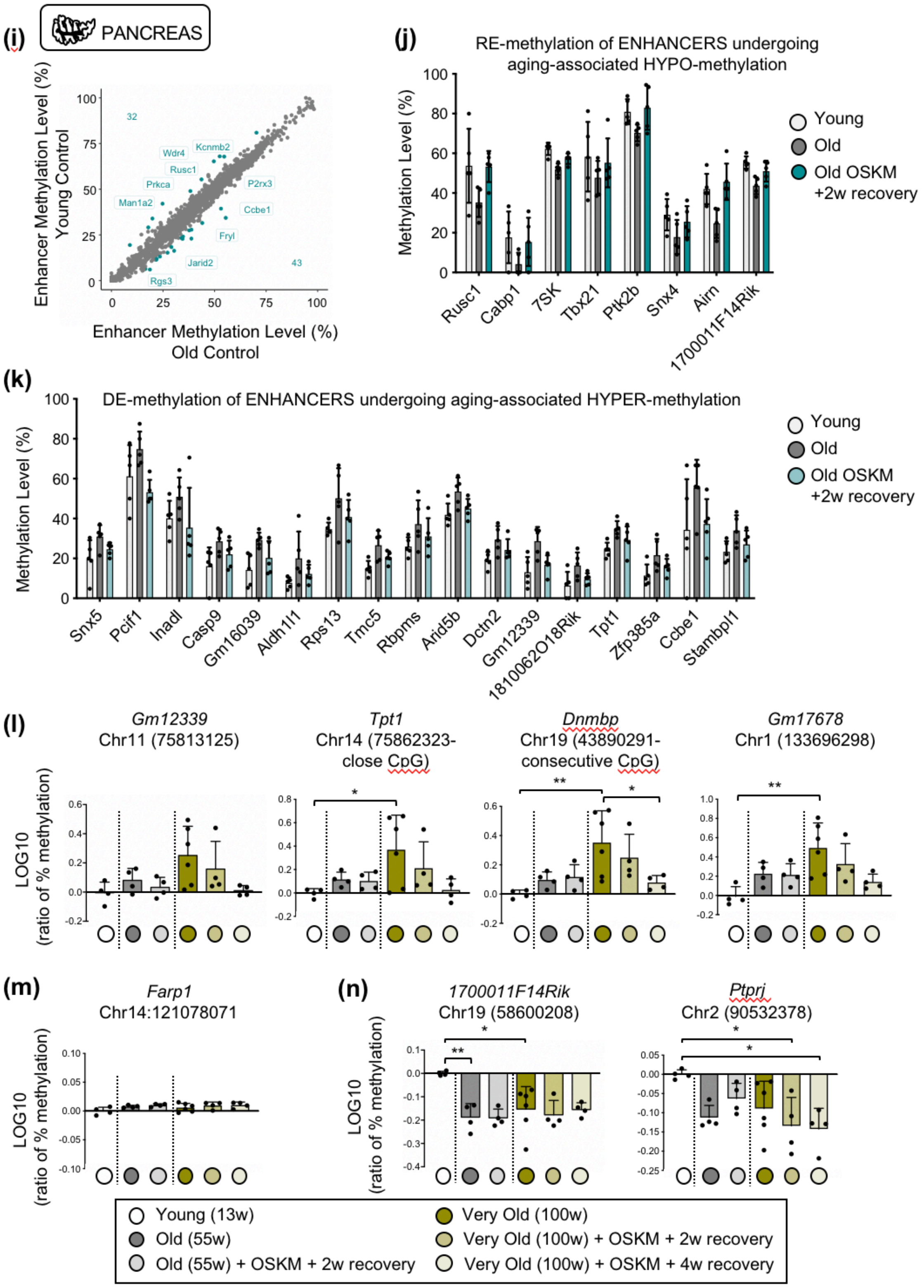

(i)
PANCREAS
Enhancer Methylation Level (%) Young Control
Enhancer Methylation Level (%) Old Control
(j)
RE-methylation of ENHANCERS undergoing aging-associated HYPO-methylation
Methylation Level (%)
Rusc1
Cabp1
7SK
Tbx21
Ptk2b
Snx4
Airn
1700011F14Rik
Young
Old
Old OSKM +2w recovery
(k)
DE-methylation of ENHANCERS undergoing aging-associated HYPER-methylation
Methylation Level (%)
Snx5
Pcif1
Inadl
Casp9
Gm16039
Aldh1l1
Rps13
Tmc5
Rbpms
Arid5b
Dctn2
Gm12339
1810062O18Rik
Tpt1
Zfp385a
Ccbe1
Stambpl1
Young
Old
Old OSKM +2w recovery
(l)
Gm12339 Chr11 (75813125)
Tpt1 Chr14 (75862323-close CpG)
Dnmbp Chr19 (43890291-consecutive CpG)
Gm17678 Chr1 (133696298)
LOG10 (ratio of % methylation)
(m)
Farp1 Chr14:121078071
LOG10 (ratio of % methylation)
(n)
1700011F14Rik Chr19 (58600208)
Ptprj Chr2 (90532378)
LOG10 (ratio of % methylation)
Young (13w)
Old (55w)
Old (55w) + OSKM + 2w recovery
Very Old (100w)
Very Old (100w) + OSKM + 2w recovery
Very Old (100w) + OSKM + 4w recovery

FIGURE S1.

Methylation profile of aging-associated differentially methylated promoters and enhancers in old-OSKM pancreas.

(a) Hematoxylin Eosin (HE) of pancreas, liver and spleen of old (55 weeks) mice treated without or with doxycycline for one week, two weeks or one week followed by two weeks of recovery. (b) RNA expression levels of pluripotency markers *Nanog*, endogenous *Oct4* (*enOct4*) and *Tfe3* in the indicated tissues (n=5 females). (c) RNA expression levels of *OSKM cassette* using *E2A-c-Myc* primers in the same tissues. (d) Immunohistochemistry of SOX2 in the pancreas of young reprogrammable mouse (10 weeks) 24 hours after intraperitoneal injection of doxycycline compared to untreated reprogrammable mouse. (e) Schematic representation of the experimental groups: young (n=5 females, reprogrammable untreated), old (n=5 females, reprogrammable untreated), old-OSKM (n=5 females, reprogrammable treated with doxycycline), very old (n=6 males and females, wild-type treated with doxycycline), very old (n=4 males and females, reprogrammable treated with doxycycline plus 2 weeks recovery), very old-OSKM (n=4 males and females, reprogrammable treated with doxycycline plus 4 weeks recovery recovery). (f) Identification of differentially methylated (DM) promoters in young *versus* old control pancreas. (g) A set of gene promoters with decreased methylation levels undergoing aging-associated hypermethylation. (h) A group of gene promoters with gain of methylation undergoing aging-associated hypomethylation. (i) Identification of differentially methylated (DM) enhancers in young *versus* old control pancreas. (j) A group of gene enhancers with gain of methylation undergoing aging-associated hypomethylation. (k) A set of gene enhancers with decreased methylation levels undergoing aging-associated hypermethylation. (l) Methylation levels of four CpGs, located in regions hypermethylated with aging measured and validated by bisulfite pyrosequencing and (m) one non-validated CpG. (n) Methylation levels measured by bisulfite pyrosequencing of two CpGs located in regions hypomethylated with aging.

Figure S2

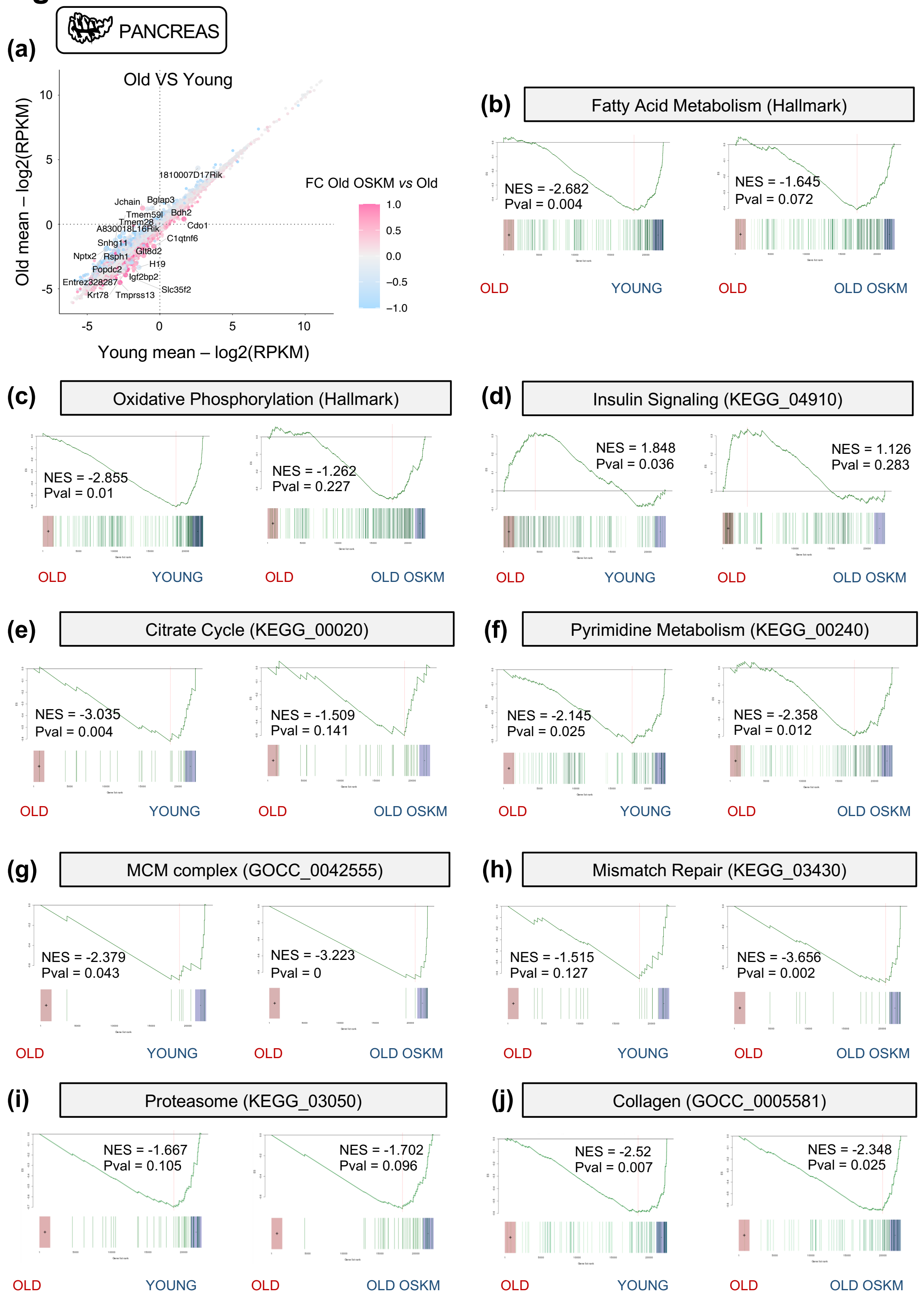
PANCREAS
(a)
Old VS Young
Old mean – log2(RPKM)
Young mean – log2(RPKM)
FC Old OSKM vs Old
1810007D17Rik
Jchain
Bglap3
Tmem59l
Bdh2
Tmem28
A830018L16Rik
Cdo1
C1qtnf6
Snhg11
Nptx2
Rsph1
Glt8d2
H19
Popdc2
Igf2bp2
Entrez328287
Slc35f2
Krt78
Tmprss13
(b)
Fatty Acid Metabolism (Hallmark)
NES = -2.682
Pval = 0.004
NES = -1.645
Pval = 0.072
OLD
YOUNG
OLD
OLD OSKM
(c)
Oxidative Phosphorylation (Hallmark)
NES = -2.855
Pval = 0.01
NES = -1.262
Pval = 0.227
OLD
YOUNG
OLD
OLD OSKM
(d)
Insulin Signaling (KEGG_04910)
NES = 1.848
Pval = 0.036
NES = 1.126
Pval = 0.283
OLD
YOUNG
OLD
OLD OSKM
(e)
Citrate Cycle (KEGG_00020)
NES = -3.035
Pval = 0.004
NES = -1.509
Pval = 0.141
OLD
YOUNG
OLD
OLD OSKM
(f)
Pyrimidine Metabolism (KEGG_00240)
NES = -2.145
Pval = 0.025
NES = -2.358
Pval = 0.012
OLD
YOUNG
OLD
OLD OSKM
(g)
MCM complex (GOCC_0042555)
NES = -2.379
Pval = 0.043
NES = -3.223
Pval = 0
OLD
YOUNG
OLD
OLD OSKM
(h)
Mismatch Repair (KEGG_03430)
NES = -1.515
Pval = 0.127
NES = -3.656
Pval = 0.002
OLD
YOUNG
OLD
OLD OSKM
(i)
Proteasome (KEGG_03050)
NES = -1.667
Pval = 0.105
NES = -1.702
Pval = 0.096
OLD
YOUNG
OLD
OLD OSKM
(j)
Collagen (GOCC_0005581)
NES = -2.52
Pval = 0.007
NES = -2.348
Pval = 0.025
OLD
YOUNG
OLD
OLD OSKM

FIGURE S2.

Transcriptional rejuvenation in old-OSKM pancreas. (a) Representation of all DEGs in old *versus* young samples colored by their alteration of expression induced by OSKM: OSKM-upregulated genes are depicted in pink and OSKM-downregulated genes are depicted in blue, while the names of the top ten genes, either upregulated or downregulated with aging, are also depicted (b) Enrichment analysis based on ROAST (Efron & Tibshirani, 2007; Wu et al., 2010) was performed comparing young *versus* old, and old *versus* old-OSKM pancreas. Old mice are 55 weeks of age. The following processes are depicted: (b) Fatty Acid Metabolism (Hallmark), (c) Oxidative Phosphorylation (Hallmark), (d) Insulin Signaling (KEGG_04910), (e) Citrate Cycle (KEGG_00020), (f) Pyrimidine Metabolism (KEGG_00620), (g) MCM complex (GOCC_0042555), (h) Mismatch Repair (KEGG_03430), (i) Proteasome (KEGG_03050), (j) Collagen (GOCC_0005581). Statistical significance was evaluated using Komolgorov-Smirnov test.

Figure S3

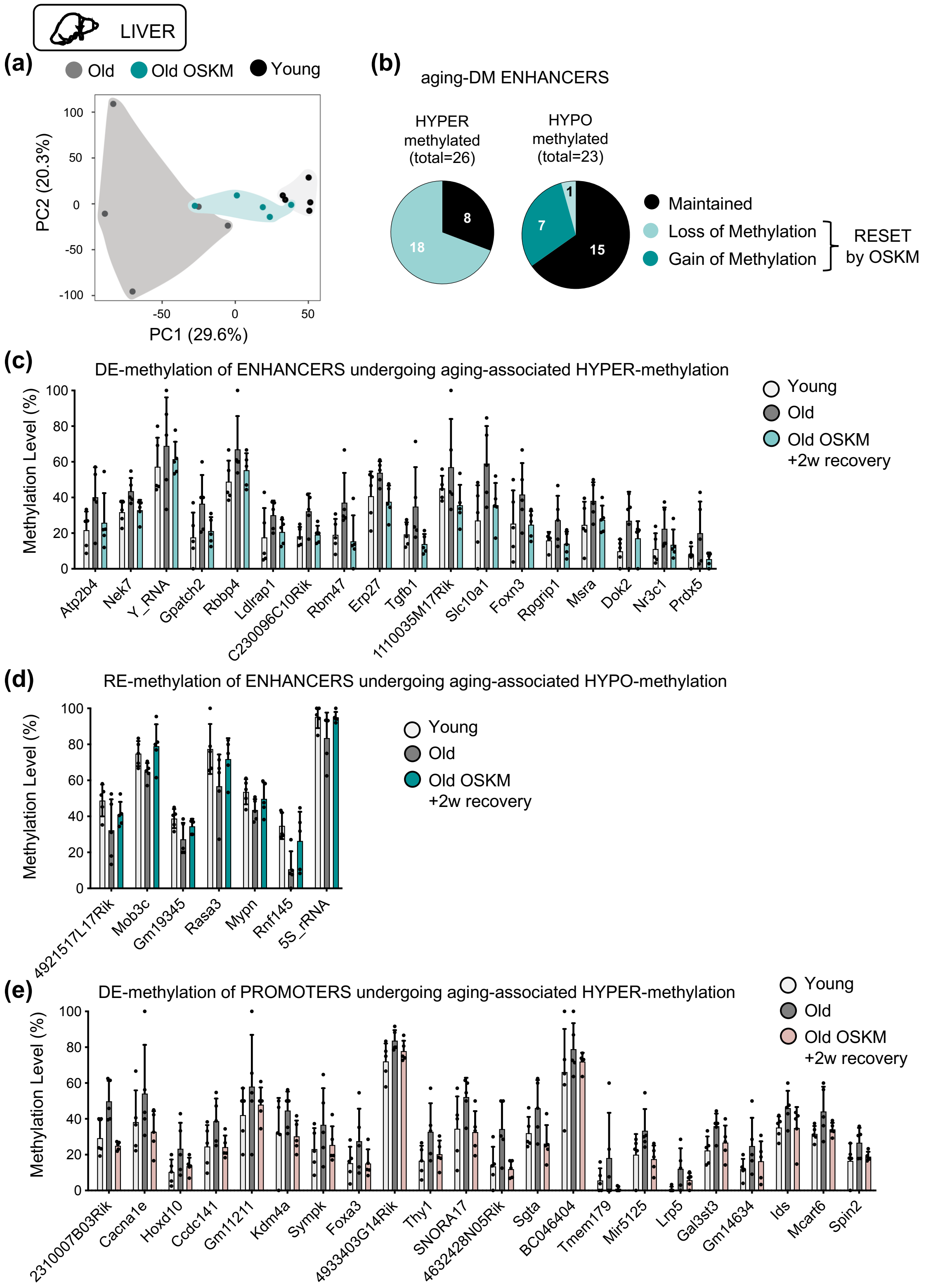
LIVER
(a)
Old
Old OSKM
Young
PC2 (20.3%)
PC1 (29.6%)
(b)
aging-DM ENHANCERS
HYPER methylated (total=26)
HYPO methylated (total=23)
8
18
1
7
15
Maintained
Loss of Methylation
Gain of Methylation
RESET by OSKM
(c)
DE-methylation of ENHANCERS undergoing aging-associated HYPER-methylation
Methylation Level (%)
Young
Old
Old OSKM +2w recovery
Atp2b4
Nek7
Y_RNA
Gpatch2
Rbbp4
Ldlrap1
C230096C10Rik
Rbm47
Erp27
Tgfb1
1110035M17Rik
Slc10a1
Foxn3
Rpgrip1
Msra
Dok2
Nr3c1
Prdx5
(d)
RE-methylation of ENHANCERS undergoing aging-associated HYPO-methylation
Methylation Level (%)
Young
Old
Old OSKM +2w recovery
4921517L17Rik
Mob3c
Gm19345
Rasa3
Mypn
Rnf145
5S_rRNA
(e)
DE-methylation of PROMOTERS undergoing aging-associated HYPER-methylation
Methylation Level (%)
Young
Old
Old OSKM +2w recovery
2310007B03Rik
Cacna1e
Hoxd10
Ccdc141
Gm11211
Kdm4a
Sympk
Foxa3
4933403G14Rik
Thy1
SNORA17
4632428N05Rik
Sgta
BC046404
Tmem179
Mir5125
Lrp5
Gal3st3
Gm14634
Ids
Mcart6
Spin2

Figure S3

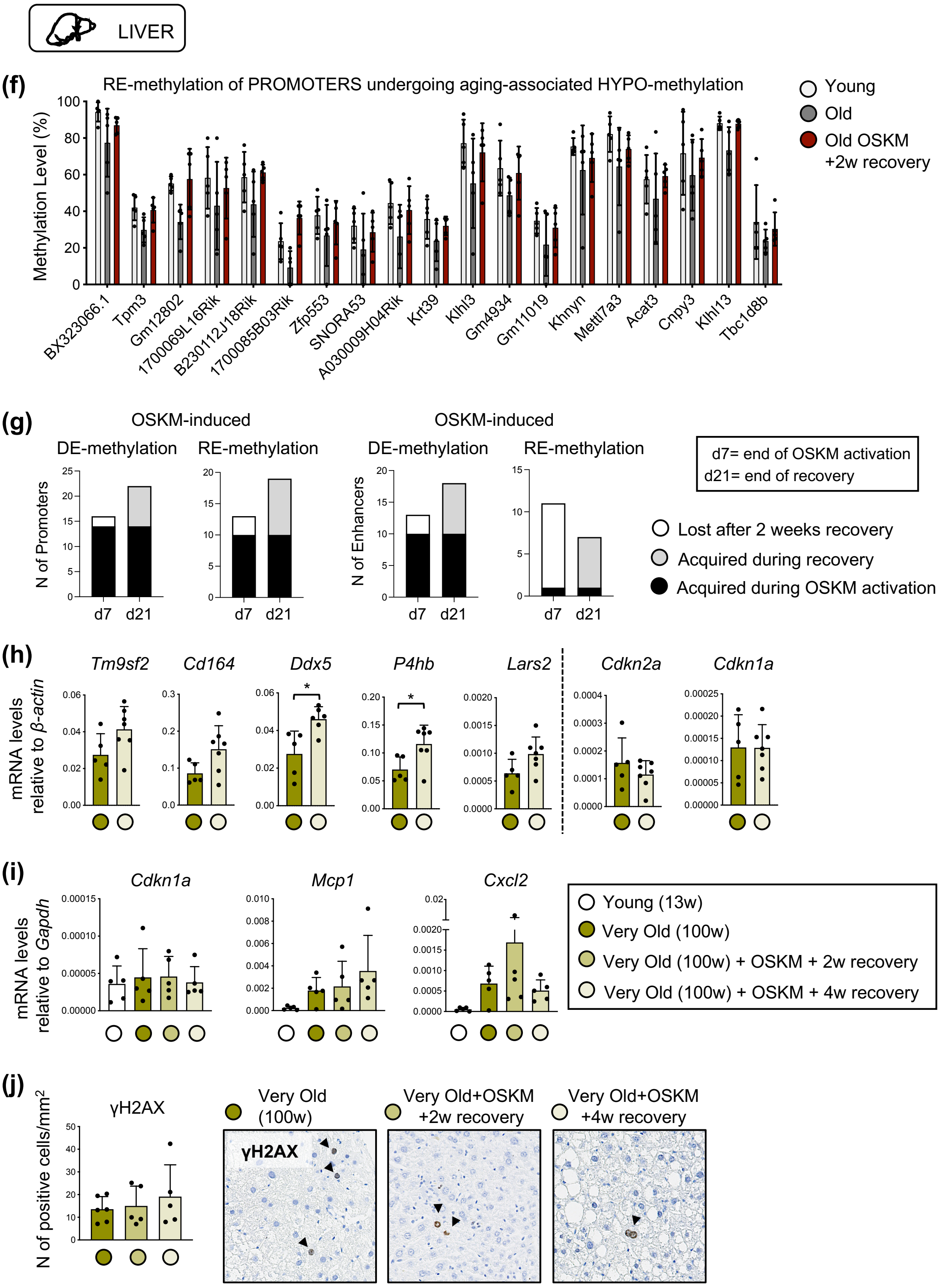

LIVER
(f)
RE-methylation of PROMOTERS undergoing aging-associated HYPO-methylation
Methylation Level (%)
Young
Old
Old OSKM +2w recovery
BX323066.1
Tpm3
Gm12802
1700069L16Rik
B230112J18Rik
1700085B03Rik
Zfp553
SNORA53
A030009H04Rik
Krt39
Klhl3
Gm4934
Gm11019
Khnyn
Mettl7a3
Acat3
Cnpy3
Klhl13
Tbc1d8b
(g)
OSKM-induced
DE-methylation
RE-methylation
N of Promoters
N of Enhancers
d7
d21
d7= end of OSKM activation
d21= end of recovery
Lost after 2 weeks recovery
Acquired during recovery
Acquired during OSKM activation
(h)
mRNA levels relative to β-actin
Tm9sf2
Cd164
Ddx5
P4hb
Lars2
Cdkn2a
Cdkn1a
(i)
mRNA levels relative to Gapdh
Cdkn1a
Mcp1
Cxcl2
Young (13w)
Very Old (100w)
Very Old (100w) + OSKM + 2w recovery
Very Old (100w) + OSKM + 4w recovery
(j)
N of positive cells/mm²
γH2AX
Very Old (100w)
Very Old+OSKM +2w recovery
Very Old+OSKM +4w recovery
γH2AX

FIGURE S3.

Old livers present rejuvenated features after transient OSKM reprogramming. (a) PCA of aging-associated DM enhancers in young, old and old-OSKM livers. Old mice are 55 weeks of age. (b) DM enhancers are classified into hyper- and hypomethylated during aging, and shown is the subset of these enhancers that alters their methylation profile due to transient OSKM activation. (c) A group of gene enhancers with gain of methylation undergoing aging-associated hypomethylation. (d) A set of gene enhancers with loss of methylation levels undergoing aging-associated hyper-methylation. (e) A set of gene promoters with decreased methylation levels after transient OSKM activation undergoing aging-associated hypermethylation in liver. (f) A group of gene promoters with gain of methylation undergoing aging-associated hypomethylation in liver. (g) The methylation status of aging-hypermethylated or hypomethylated promoters and enhancers that were found above to be OSKM-demethylated or remethylated respectively was evaluated directly after OSKM cessation (day 0 post-recovery) and 14 days post-recovery in the liver samples. (h) The expression of global aging genes identified by mouse Aging Cell Atlas(Zhang et al., 2021), as well as p16 (*Cdkn2a)* and p21 (*Cdkn1a)* expression was evaluated in very old livers (100 weeks; group 2 consists of 5 wild-type mice as control and 7 reprogrammable mice activating OSKM for 1 week and 4 weeks of recovery). (i) p21 (*Cdkn1a*), *Mcp1* and *Cxcl2* expression in the liver of young (13 weeks) and very old (100 weeks; group 1) mice. (j) Immunohistochemistry of γH2AX in the liver of very old (100 weeks) mice. Statistical significance was evaluated using one-way ANOVA with Tukey's multiple comparison method, and comparisons are indicated as *$P < 0.05$, **$P < 0.01$ and ***$P < 0.001$. Bars in c-f and h-k represent the standard deviation (SD) of the data.

Figure S4

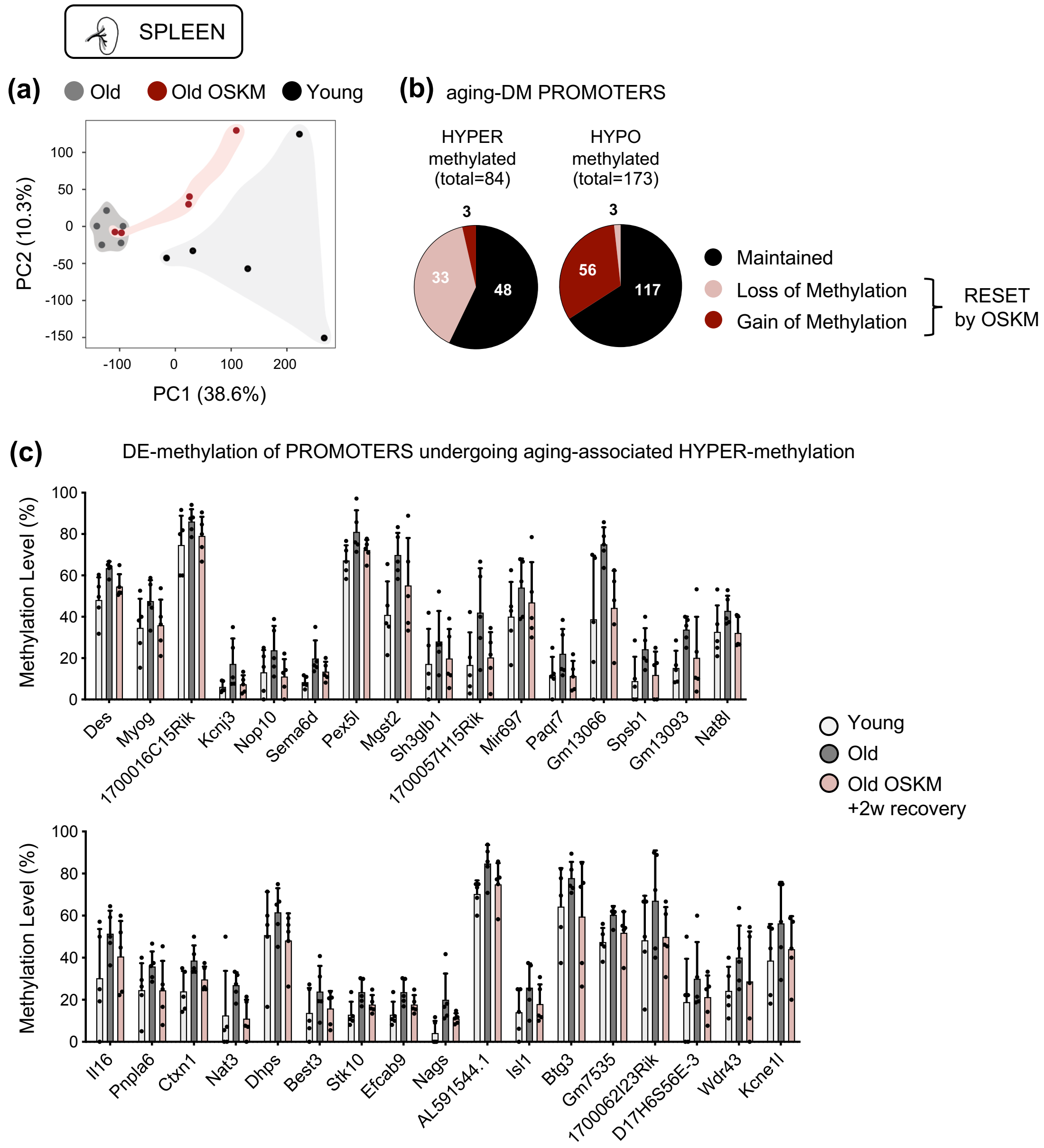
SPLEEN
(a)
Old
Old OSKM
Young
PC2 (10.3%)
PC1 (38.6%)
(b)
aging-DM PROMOTERS
HYPER methylated (total=84)
HYPO methylated (total=173)
3
33
48
3
56
117
Maintained
Loss of Methylation
Gain of Methylation
RESET by OSKM
(c)
DE-methylation of PROMOTERS undergoing aging-associated HYPER-methylation
Methylation Level (%)
Des
Myog
1700016C15Rik
Kcnj3
Nop10
Sema6d
Pex5l
Mgst2
Sh3glb1
1700057H15Rik
Mir697
Paqr7
Gm13066
Spsb1
Gm13093
Nat8l
Young
Old
Old OSKM +2w recovery
Il16
Pnpla6
Ctxn1
Nat3
Dhps
Best3
Stk10
Efcab9
Nags
AL591544.1
Isl1
Btg3
Gm7535
1700062I23Rik
D17H6S56E-3
Wdr43
Kcne1l

Figure S4

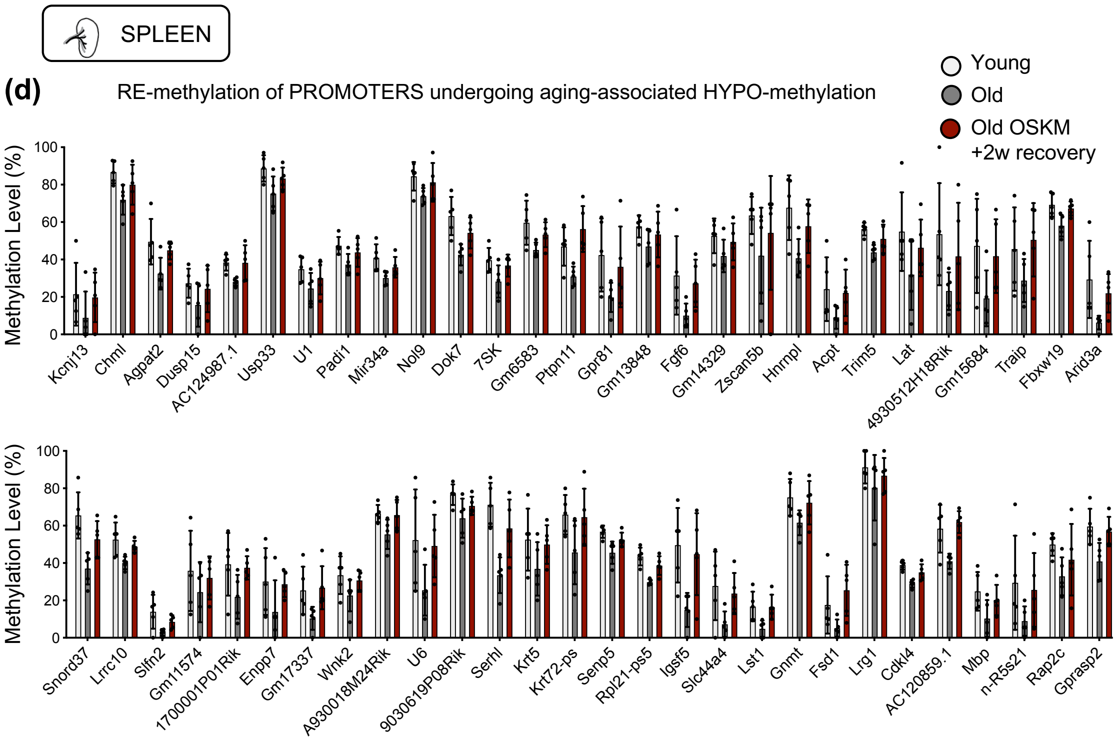

SPLEEN
(d)
RE-methylation of PROMOTERS undergoing aging-associated HYPO-methylation
Young
Old
Old OSKM +2w recovery
Methylation Level (%)
Kcnj13
Chml
Agpat2
Dusp15
AC124987.1
Usp33
U1
Padi1
Mir34a
Nol9
Dok7
7SK
Gm6583
Ptpn11
Gpr81
Gm13848
Fgf6
Gm14329
Zscan5b
Hnrnpl
Acpt
Trim5
Lat
4930512H18Rik
Gm15684
Traip
Fbxw19
Arid3a
Snord37
Lrrc10
Slfn2
Gm11574
1700001P01Rik
Enpp7
Gm17337
Wnk2
A930018M24Rik
U6
9030619P08Rik
Serhl
Krt5
Krt72-ps
Senp5
Rpl21-ps5
Igsf5
Slc44a4
Lst1
Gnmt
Fsd1
Lrg1
Cdkl4
AC120859.1
Mbp
n-R5s21
Rap2c
Gprasp2

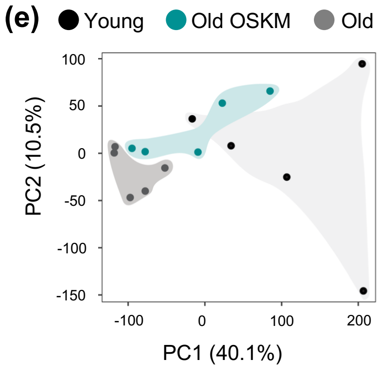

(e)
Young
Old OSKM
Old
PC2 (10.5%)
PC1 (40.1%)

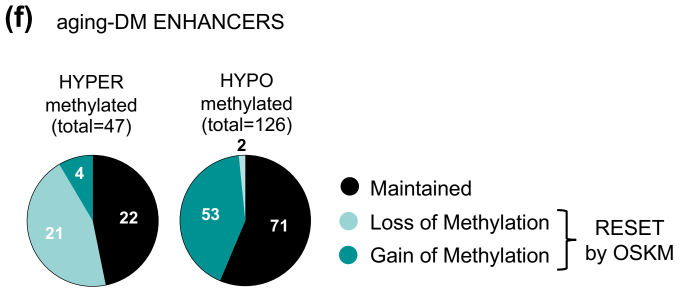

(f)
aging-DM ENHANCERS
HYPER methylated (total=47)
HYPO methylated (total=126)
4
22
21
2
53
71
Maintained
Loss of Methylation
Gain of Methylation
RESET by OSKM

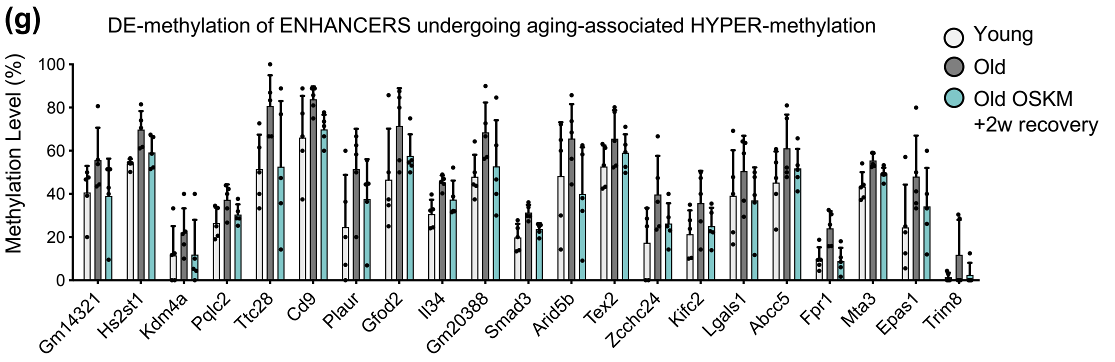

(g)
DE-methylation of ENHANCERS undergoing aging-associated HYPER-methylation
Young
Old
Old OSKM +2w recovery
Methylation Level (%)
Gm14321
Hs2st1
Kdm4a
Pqlc2
Ttc28
Cd9
Plaur
Gfod2
Il34
Gm20388
Smad3
Arid5b
Tex2
Zcchc24
Kifc2
Lgals1
Abcc5
Fpr1
Mta3
Epas1
Trim8

Figure S4

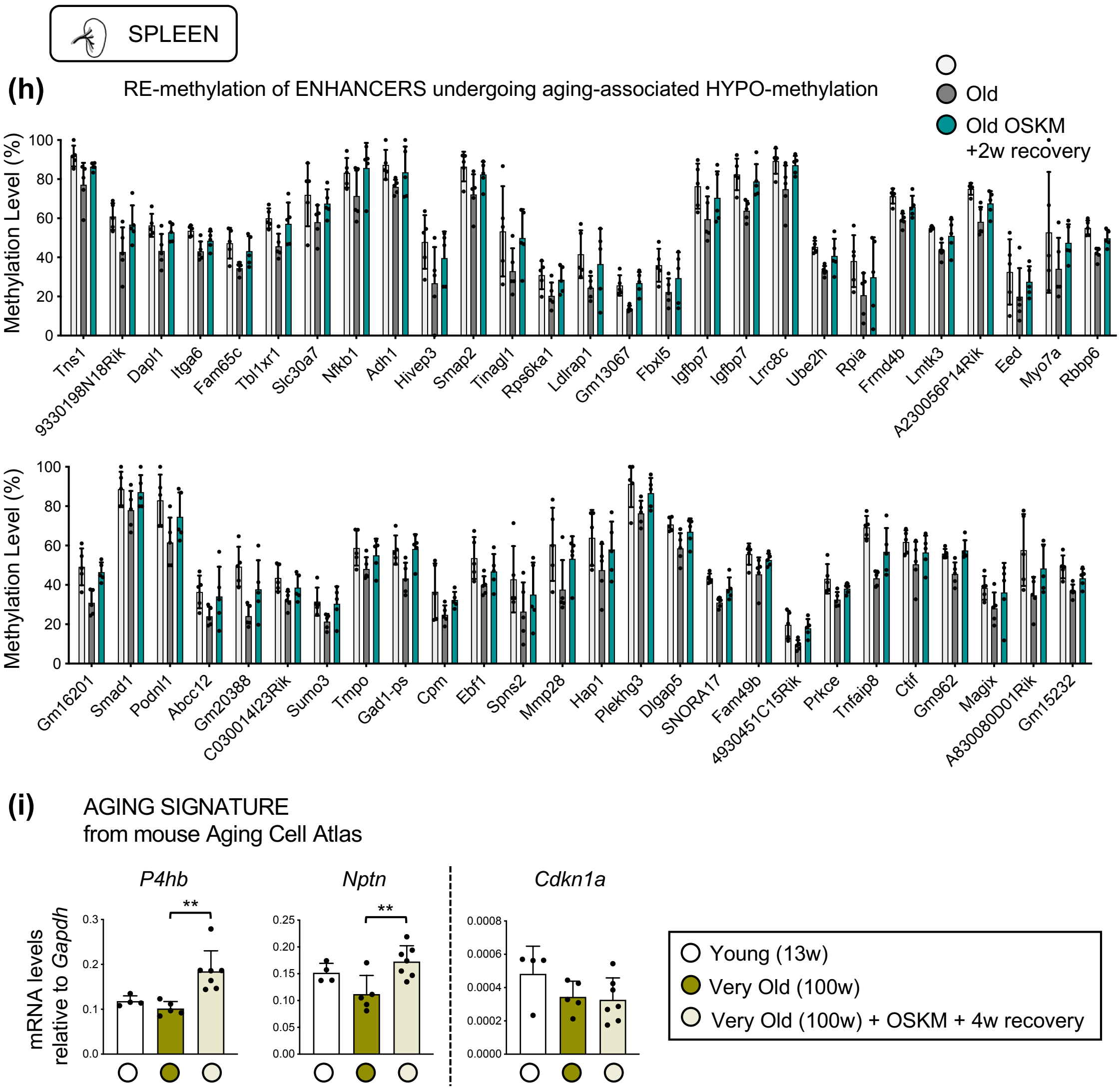

SPLEEN
(h)
RE-methylation of ENHANCERS undergoing aging-associated HYPO-methylation
Old
Old OSKM +2w recovery
Methylation Level (%)
0
20
40
60
80
100
Tns1
9330198N18Rik
Dapl1
Itga6
Fam65c
Tbl1xr1
Slc30a7
Nfkb1
Adh1
Hivep3
Smap2
Tinagl1
Rps6ka1
Ldlrap1
Gm13067
Fbxl5
Igfbp7
Igfbp7
Lrrc8c
Ube2h
Rpia
Frmd4b
Lmtk3
A230056P14Rik
Eed
Myo7a
Rbbp6
Gm16201
Smad1
Podnl1
Abcc12
Gm20388
C030014I23Rik
Sumo3
Tmpo
Gad1-ps
Cpm
Ebf1
Spns2
Mmp28
Hap1
Plekhg3
Dlgap5
SNORA17
Fam49b
4930451C15Rik
Prkce
Tnfaip8
Ctif
Gm962
Magix
A830080D01Rik
Gm15232
(i)
AGING SIGNATURE
from mouse Aging Cell Atlas
P4hb
Nptn
Cdkn1a
mRNA levels relative to Gapdh
**
**
Young (13w)
Very Old (100w)
Very Old (100w) + OSKM + 4w recovery

Figure S4.

Evidences of OSKM-induced rejuvenation in haemopoietic cells. (a) Principal Component Analysis (PCA) of aging-related differentially methylated (DM) promoters of young, old and old-OSKM spleens. (b) DM promoters are classified into hyper- and hypo-methylated during aging, and shown is the number of these promoters that alter their methylation profile due to transient OSKM activation. (c) A set of gene promoters with decreased methylation levels after transient OSKM activation undergoing aging-associated hypermethylation in spleen. (d) A group of gene promoters with gain of methylation undergoing aging-associated hypomethylation in spleen. (e) PCA of aging-associated DM enhancers of young, old and old-OSKM spleen. (f) DM enhancers are classified into hyper- and hypo-methylated during aging, and shown is the subset of these enhancers that alters their methylation profile due to transient OSKM activation. (g) A group of gene enhancers with loss of methylation undergoing aging-associated hypermethylation. (h) A set of gene enhancers with gain of methylation undergoing aging-associated hypomethylation. (i) The expression of global aging genes identified by mouse Aging Cell Atlas (Zhang et al., 2021), as well as p21 (*Cdkn1a)* expression was evaluated in very old spleens (100 weeks; 5 wild-type mice as control, 7 reprogrammable mice activating OSKM for 1 week and recovering for 4 weeks) compared to young (13 weeks; n= 4) control spleens. Bars in c-d and g-i represent the standard deviation (SD) of the data.

Figure S5

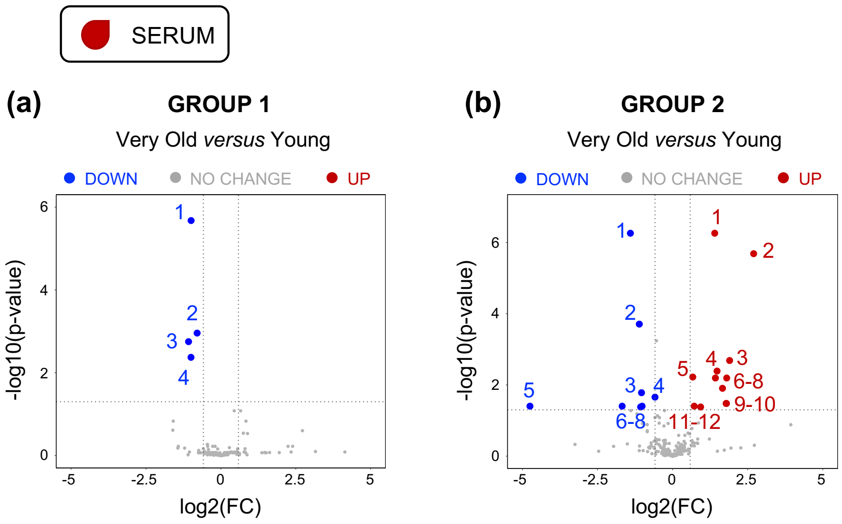

1. Thymine
2. Ketoisocaproic acid
3. Trimethyl-lysine
4. 4-hydroxyproline

1. 4-hydroxyproline
2. 2-hydroxy-3-methylbutyric acid
3. Glycine
4. Nonadecenoic acid
5. Inosine
6. 3-methylglutarylcarnitine
7. UMP
8. Succinyladenosine

1. 3-Phenyllactic acid
2. Xylose
3. Indole-3-acrylic acid
4. Arabinose
5. Arabitol
6. Indole-3-acetic acid
7. Indole-3-lactic acid
8. Quinolinic acid
9. Indole-3-propionic acid
10. Methyl-indole-3-acetic acid
11. 5-Hydroxy-DL-tryptophan
12. N-Acetyl-DL-tryptophan

FIGURE S5. Metabolomic analysis in the serum of very old-OSKM mice. (a) Volcano plot depicting the differentially present metabolites (fold change>1.5 and adjusted pval<0.05) in the serum of very old (100 weeks, n=6) *versus* young (13 weeks, n=3) female mice as *Group 1*, and (b) as *Group 2* consisting of very old mice (100 weeks, n=6) versus young (15 weeks, n=5) female mice. Independent metabolomic analyses have been performed for the two different cohorts of mice. Statistical significance was evaluated using a non-parametric linear mixed-effect model. Comparisons are indicated as *P < 0.05, **P < 0.01 and ***P < 0.001.
